# Supplementary figures and images for: Quantitative analysis of visual codewords of a protein distance matrix
Source: PLoS One. 2022 Feb 4;17(2):e0263566. doi: 10.1371/journal.pone.0263566 (PMC8815937; doi:10.1371/journal.pone.0263566)

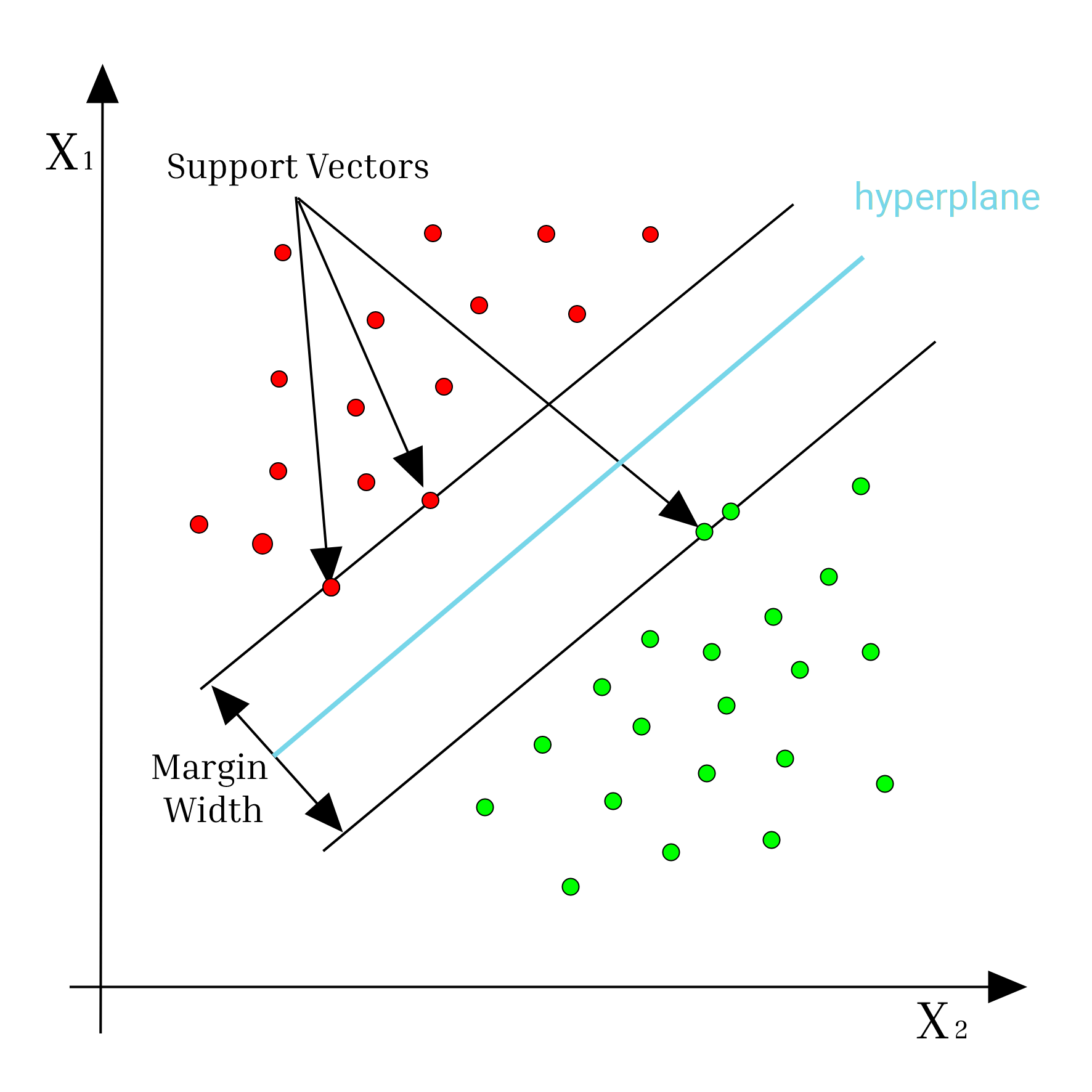

Supplement: S1 Fig — The hyperplane is a function that separates features into multiple classes. The function that separates features in 2D space is a line, while in 3D space it is a plane. When more dimensions are introduced, this function is called a hyperplane. The figure shows two classes of data in 2D space, two support vectors, margin width, and a hyperplane, which in this case is a line. (TIF) [file pone.0263566.s001.tif]

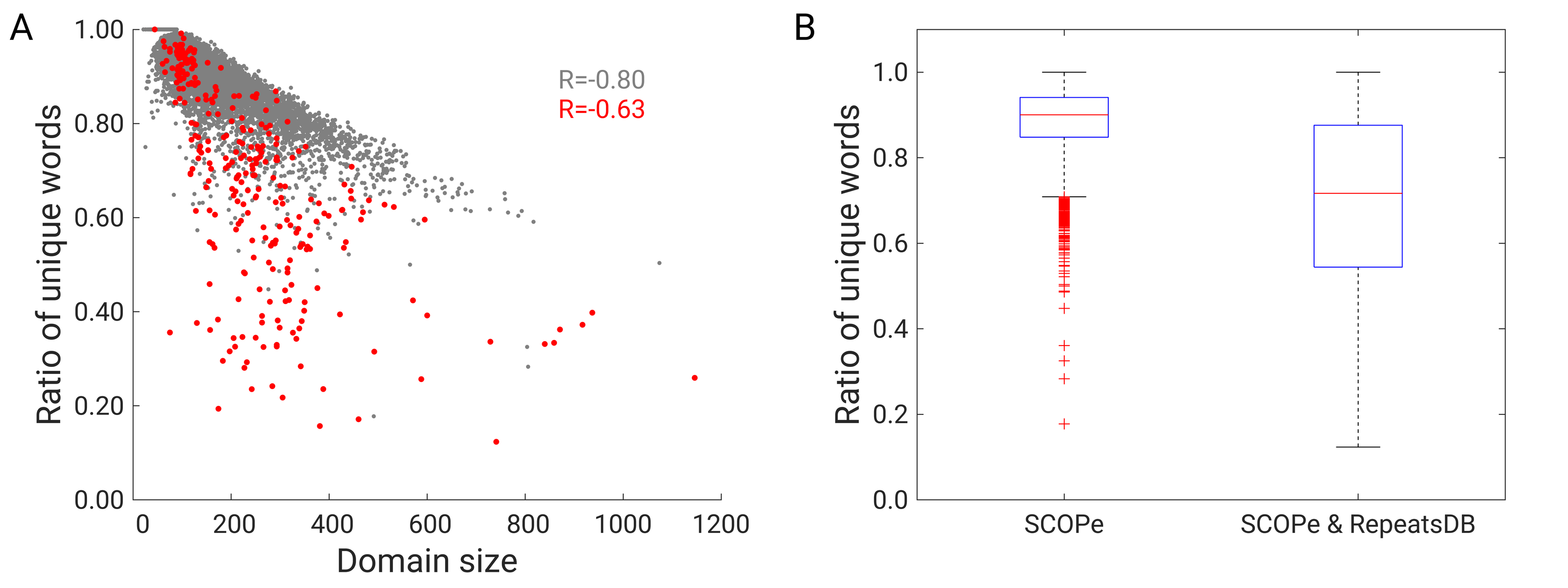

Supplement: S2 Fig — (A) Ratio of unique words against the number of features. Domains that are members of RepeatsDB and our dataset data set are marked with red dots. (B) The ratios of unique codewords from SCOPe domains (not members of RepeatsDB) and SCOPe domains that are members of RepeatsDB. (TIF) [file pone.0263566.s002.tif]

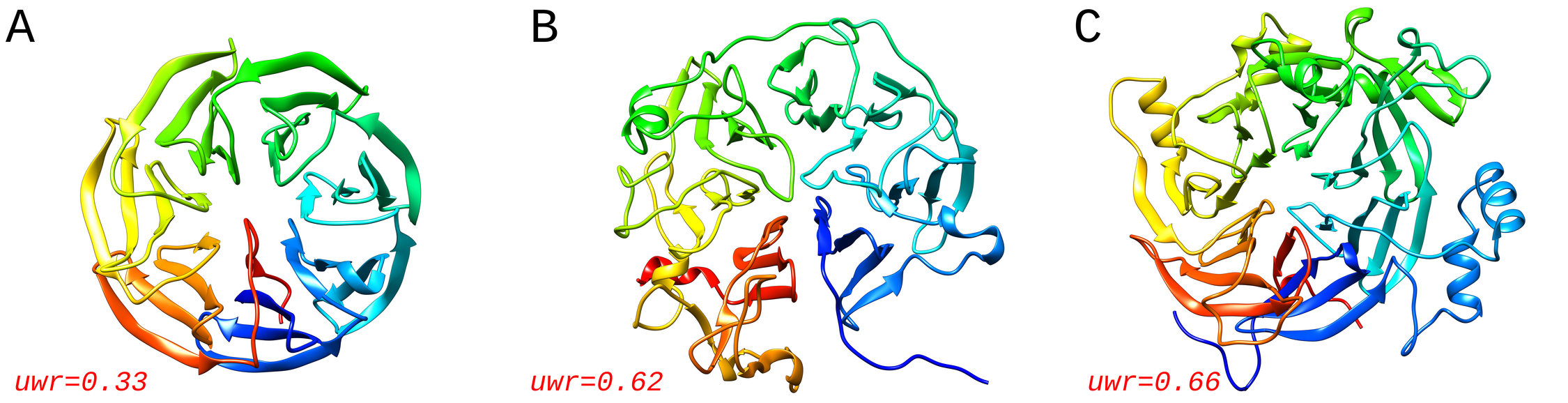

Supplement: S3 Fig — The domains are sorted by the ratio of unique words from lowest (left) to highest (right). (A) YVTN repeat (SCOPe: d1l0qa2) has unique word ratio uwr = 0.33, (B) domain of oligoxyloglucan reducing end-specific cellobiohydrolase (SCOPe: d2ebsa1) has unique word ratio uwr = 0.62 and (C) domain of oligoxyloglucan reducing end-specific cellobiohydrolase (SCOPe: d2iwka1) has unique word ratio uwr = 0.66. (TIF) [file pone.0263566.s003.tif]
