# Supplementary material for: Quantitative analysis of visual codewords of a protein distance matrix
Source: PLoS One. 2022 Feb 4;17(2):e0263566. doi: 10.1371/journal.pone.0263566 (PMC8815937; doi:10.1371/journal.pone.0263566)
Supplement: S1 Table — The SCOPe entries were taken from SCOPe 2.07 and are less than 40% identical. The domains are sorted by the ratio of unique words from lowest to highest. (DOCX) [file pone.0263566.s004.docx]

**S1 Table.** Unique word ratio for the SCOPe fold class 7-bladed beta-propeller (b.69), which contains 15 superfamilies. The SCOPe entries were taken from SCOPe 2.07 and are less than 40% identical. The domains are sorted by the ratio of unique words from lowest to highest.

| **SCOPe ID** | **Superfamily** | **Ratio** |
| --- | --- | --- |
| d1l0qa2 | b.69.2 | 0.33 |
| d3qhyb_ | b.69.5 | 0.39 |
| d1erja_ | b.69.4 | 0.42 |
| d1ri6a1 | b.69.11 | 0.42 |
| d1yfqa1 | b.69.4 | 0.48 |
| d1jofa_ | b.69.10 | 0.52 |
| d2vdra_ | b.69.8 | 0.52 |
| d5n4fa2 | b.69.7 | 0.52 |
| d1k3ia3 | b.69.1 | 0.53 |
| d2b5la1 | b.69.4 | 0.54 |
| d2hu7a1 | b.69.7 | 0.54 |
| d1a12a_ | b.69.5 | 0.55 |
| d1mdah_ | b.69.2 | 0.58 |
| d1xksa_ | b.69.14 | 0.59 |
| d1k32a3 | b.69.9 | 0.60 |
| d1jmxb_ | b.69.2 | 0.60 |
| d2xzga1 | b.69.6 | 0.61 |
| d2ebsa1 | b.69.13 | 0.62 |
| d3fsna_ | b.69.15 | 0.63 |
| d1olza2 | b.69.12 | 0.65 |
| d2iwka1 | b.69.3 | 0.66 |
