# Supplementary material for: Quantitative analysis of visual codewords of a protein distance matrix
Source: PLoS One. 2022 Feb 4;17(2):e0263566. doi: 10.1371/journal.pone.0263566 (PMC8815937; doi:10.1371/journal.pone.0263566)
Supplement: S1 Dataset — The supplemental data includes distance matrix images (grayscale, PNG format) and a Matlab script. (DOC) [file pone.0263566.s005.doc]

Dataset is available at open-access repository ZENODO.

URL:

<https://zenodo.org/record/5906637>

DOI: 10.5281/zenodo.5906637
